# A User-Centered Chatbot (Wakamola) to Collect Linked Data in Population Networks to Support Studies of Overweight and Obesity Causes: Design and Pilot Study

**DOI:** 10.2196/17503

**Published:** 2021-04-14

**Authors:** Sabina Asensio-Cuesta, Vicent Blanes-Selva, J Alberto Conejero, Ana Frigola, Manuel G Portolés, Juan Francisco Merino-Torres, Matilde Rubio Almanza, Shabbir Syed-Abdul, Yu-Chuan (Jack) Li, Ruth Vilar-Mateo, Luis Fernandez-Luque, Juan M García-Gómez

**Affiliations:** 1 Instituto de Tecnologías de la Información y Comunicaciones Universitat Politècnica de València Valencia Spain; 2 Instituto Universitario de Matemática Pura y Aplicada Universitat Politècnica de València Valencia Spain; 3 Department of Nutrition and Food Science Universitat de València Valencia Spain; 4 Department of Endocrinology and Nutrition Hospital La Fe Universitat de València Valencia Spain; 5 International Center for Health Information Technology Taipei Medical University Taipei Taiwan; 6 Unidad Mixta de Tic aplicadas a la reingeniería de procesos socio-sanitarios Instituto de Investigación Sanitaria La Fe Valencia Spain; 7 Adhera Health Inc Palo Alto, CA United States

**Keywords:** mHealth, obesity, overweight, chatbot, assessment, public health, Telegram, user-centered design, Social Network Analysis

## Abstract

**Background:**

Obesity and overweight are a serious health problem worldwide with multiple and connected causes. Simultaneously, chatbots are becoming increasingly popular as a way to interact with users in mobile health apps.

**Objective:**

This study reports the user-centered design and feasibility study of a chatbot to collect linked data to support the study of individual and social overweight and obesity causes in populations.

**Methods:**

We first studied the users’ needs and gathered users’ graphical preferences through an open survey on 52 wireframes designed by 150 design students; it also included questions about sociodemographics, diet and activity habits, the need for overweight and obesity apps, and desired functionality. We also interviewed an expert panel. We then designed and developed a chatbot. Finally, we conducted a pilot study to test feasibility.

**Results:**

We collected 452 answers to the survey and interviewed 4 specialists. Based on this research, we developed a Telegram chatbot named Wakamola structured in six sections: personal, diet, physical activity, social network, user's status score, and project information. We defined a user's status score as a normalized sum (0-100) of scores about diet (frequency of eating 50 foods), physical activity, BMI, and social network. We performed a pilot to evaluate the chatbot implementation among 85 healthy volunteers. Of 74 participants who completed all sections, we found 8 underweight people (11%), 5 overweight people (7%), and no obesity cases. The mean BMI was 21.4 kg/m^2^ (normal weight). The most consumed foods were olive oil, milk and derivatives, cereals, vegetables, and fruits. People walked 10 minutes on 5.8 days per week, slept 7.02 hours per day, and were sitting 30.57 hours per week. Moreover, we were able to create a social network with 74 users, 178 relations, and 12 communities.

**Conclusions:**

The Telegram chatbot Wakamola is a feasible tool to collect data from a population about sociodemographics, diet patterns, physical activity, BMI, and specific diseases. Besides, the chatbot allows the connection of users in a social network to study overweight and obesity causes from both individual and social perspectives.

## Introduction

The percentage of overweight people has not stopped increasing worldwide since the 1980s [1]. In the United States, more than two-thirds of adults and nearly one-third of children and youth are overweight or obese [2]. According to the World Health Organization, in Europe, more than 50% of the population is overweight, and 20% is obese [3].

Obesity is a complex problem with individual, socioeconomic, and environmental factors [4]. From a social perspective, Fowler and Christakis conducted a study about the spread of obesity in a large social network over 32 years (Framingham Heart Study) [5] and found evidence of the “contagion” of obesity among people in close social circles. Indeed, the relevant finding in the study suggests that ties between friends have an even more significant effect on a person's risk of obesity than genes. A person's chances of becoming obese increased by 57% if he or she had a friend who became obese in a given interval. Moreover, for a wide variety of conditions and networks, Bahr et al [6] showed that individuals with similar BMIs would cluster together into groups.

Furthermore, chatbots, also referred to as conversational user interfaces, are gradually being adopted in mobile health (mHealth) apps [7] and serve to assess the long-term user experience [8]. A chatbot is a conversation platform that interacts with users via a chatting interface. Since its use can be facilitated by linkages with the major social network service messengers (eg, WhatsApp, Telegram), general users can easily access and receive various health services [9]. Laranjo et al [7] provide an overview of research related to conversational user interfaces in health care.

Previous studies suggest that chatbots may have the potential to contribute to obesity and overweight prevention and management [10]. In 2017, Kowatsch et al [11] designed a text-based health care chatbot to effectively support patients and health professionals in therapeutic settings beyond on-site consultations and applied to childhood obesity control. In 2018, Huang et al [12] developed a chatbot integrated in the SWITCHes app, where the chatbot helps monitor users’ health; users also talk to the chatbot and get information in a real-time manner or take a bot’s advice, including diet and exercise plans, in the context of healthy recommendation. In 2018, Holmes et al [10] described the design and development of a chatbot (WeightMentor), a self-help motivational tool for weight loss maintenance. In 2019, Stephens et al [13] implemented a behavioral coaching chatbot (Tess) to help support teens in a weight management program. However, chatbots can be useful not only in obesity control, monitoring, and promotion of healthy habits, as mentioned in these studies, but also as effective tools to collect data in large populations to study obesity causes and lead prevention. So far, face-to-face or online questionnaires are widely used to collect data directly from people about their weight, diet, and physical activity habits [14-17]. However, recent studies indicate that chatbots may be more attractive to users than classic questionnaires because people associate them with entertainment, social, and relational factors [8,18]. In addition, users have curiosity about what they view as a novel phenomenon [19].

Moreover, chatbots also enable the development of gamification strategies that can have a positive impact on health and wellbeing [20,21], already widely applied to online surveys [22]. Hamari defines gamification as “a process of enhancing services with (motivational) affordances in order to invoke gameful experiences and further behavioral outcomes” [23]. Concerning game mechanics, feedback and socialization aspects are recurrently employed to gamify eHealth. Social features, rewards, and progress tracking are powerful mechanics for producing positive effects on users [24]. Focusing on gamification strategies applied in chatbots, chatbots are able to implement mobile app stickiness strategies to improve user engagement, such as gaming, dexterity, responsiveness and feedback after coming in contact with the app, ease of figuring out how to operate the app, forums, multimedia display, and emotional engagement [25,26]. Siutila [27] identified tracking options in popular chatbots [28-30]: a system of points, leaderboards, achievements/badges, levels, story/theme, clear goals, feedback, rewards, progress, and challenge. 

This study reports the user-centered design and feasibility study of a chatbot to collect linked data about diet, physical activity, weight, obesity risk, living area, and social network, to support research regarding individuals and social causes of obesity and overweight. Here, we describe the user-centered approach applied in the design and development of the chatbot. We also present a pilot study to test the chatbot’s feasibility. 

## Methods

### Ethics

Ethical approval was obtained for this study from the Ethical Committee of the Universitat Politècnica de València (UPV; Ethical Code: P7_12_11_2018).

### Users’ Needs Investigation

Applying a user-centered approach, we started the design of the chatbot by collecting potential users’ expectations and preferences. We briefly expose the three parts into which we split the information collection: (1) a survey about interests and expectations, (2) an analysis of graphical preferences, and (3) a specialist panel’s advice on the medical content. Further details of each of these parts can be found in Multimedia Appendix 1 [5,6,18,20,21,23-69].

First, a survey was designed including questions about sociodemographic data, self-perception of overweight, diet and physical activity, favorite colors for the app’s purpose, the potential utility of the app, future use of the app, type of preferred diffusion, and desired functionalities. The survey included 13 questions in total.

Second, to investigate user preferences regarding the graphical features of the interface, wireframes were designed by design students and included in the survey to be scored on a 1 to 5 scale. Wireframes were designed following these general specifications: the appearance of the app breaks with the stigma of obesity and overweight and motivates its use; the app promotes a healthy lifestyle; the elements to be designed for each alternative were the chatbot’s name, launch icon, splash, and main menu screen with preliminary options such as user’s personal data, calculating risk, and suggesting healthy activities. To design the wireframes, students reviewed mHealth apps in the obesity and overweight field. No limitations were specified for the graphical or aesthetic features. To define the chatbot’s colors, we completed the survey questions with research about current evidence regarding colors and their effects on people’s feelings [31,32]. 

Third, we also formed an expert panel composed of 1 nutritionist and 3 clinicians, all of whom were endocrine specialists. After a project introduction, we addressed the panel with three research questions: (1) what data would be relevant for study of obesity and overweight, according to current knowledge, (2) if there are validated questionnaires to get these data, and (3) how obesity and overweight risk of a user could be assessed.

### Chatbot’s Design and Development

**Figure 1:**
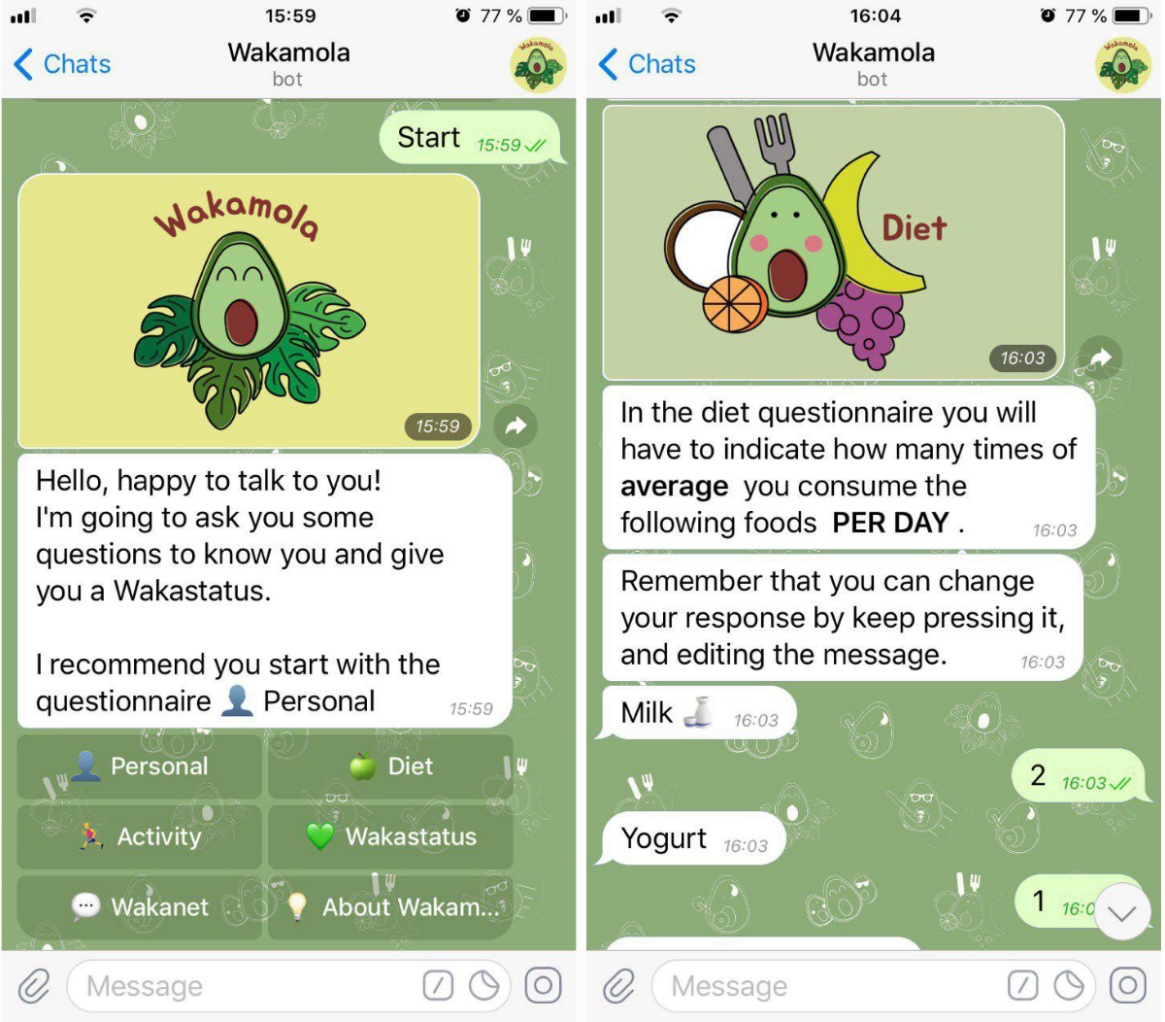
Screenshot of Wakamola’s main menu and diet section.

Based on the users’ survey and expert criteria involved in the study, we decided to include six sections in the chatbot: Personal, Diet, physical activity habits (Activity), social network (Wakanet), status (Wakastatus), and project information (About Wakamola) (Figure 1; Multimedia Appendix 2). “Personal,” “Diet,” and “Activity” were interactive surveys based on standardized questionnaires, whereas “social network” implemented a sharing mechanism based on a sticky strategy. More importantly, we defined a novel user status assessment named Wakastatus. It was a gamified version of an obesity risk assessment based on diet, physical activity, and neighborhood (relationships) status to give feedback and motivation to the user. Moreover, the word “risk” was changed to “status” to provide a positive message for the user’s obesity and overweight assessment. Further information about gamification elements in Wakamola are available in Table 1 and Multimedia Appendix 1.

**Table 1:** Gamification strategies implemented in Wakamola.

Gamification strategy	Implementation in Wakamola
System of points (scores); goals	Wakamola scores on a scale of 0 to 100 (the higher the better, goal 100) Global status score (Wakastatus) Diet score (Wakalimentation) Activity score (Activity) BMI score (WakaBMI) Social network score (Wakasocial)
Socialization	Wakamola’s social network
Feedback to the user	Self-assessment of overweight and obesity risk: Wakamola’s scores, BMI category, and level of obesity risk User’s network graphical representation, BMI/Wakastatus shown inside nodes, colors based on BMI/Wakastatus category
Emotional engagement	Personification of the chatbot through the Wakamola character Introduction of humanlike cues in Wakamola chatbot to increase users’ emotional connection [18] (eyes, mouth, expressions of effort and happiness) Use of emoji added to the Wakamola’s text messages to create a more realistic and friendly conversation [35]

The Personal section includes 16 questions about weight, height, gender, age, level of education, marital status, how many people are at home, main activity (ie, study or work), zip code, sleep hours, and cigarette consumption. In addition, the chatbot asks if the user has ever received a diagnosis or is taking medication for hypertension, diabetes, high cholesterol, or cardiovascular disease. The clinicians defined these questions for further analysis regarding overweight and obesity factors.

Questions in the Diet section were adapted from the “Short questionnaire on frequency of dietary intake” [33]. In total, 51 questions regarding food types (items) and consumption frequencies are included. Diet question responses (items) were scored based on the “Spanish diet quality according to the healthy eating index,” with items’ scores from 1 to 10 (the higher the score of the item, the less healthy its consumption) [70] (Tables S1 and S2 in Multimedia Appendix 1).

In the Activity section with 7 questions, the short form of the International Physical Activity Questionnaire (IPAQ) has been applied to define the chatbot’s questions and scoring. This IPAQ version is recommended, especially when the object of investigation is population monitoring [71].

The Wakanet section has been developed to share the Wakamola chatbot between contacts, following a sticky strategy. This is how the users’ social networks and subnetworks are created to further analysis about how their social relations and habits could influence or be influenced from an overweight and obesity perspective. This section first shows a message with the user’s total contacts, broken down by house, family, friends, and work contacts. Four different invitations are then created as chatbot messages to be shared with the target group of contacts: (1) people the user lives with (home), (2) friends, (3) family, and (4) work contacts. This section implements the community gamification strategy in the chatbot.

The Wakastatus section shows a normalized score calculated from previous data collected in the personal, diet, activity, and social network sections and normalized between 0 and 100; the higher the better. The Diet score is the sum of scores for each food and its consumption frequency; this score is also normalized between 0 and 100. The Activity score is calculated according to the short form of the IPAQ [71]. This result is normalized between 0 to 100 to present the final Activity score.

Additionally, we calculate the BMI score (WakaBMI) from the Personal section (weight and height), obtaining 100 points for normal weight (18.5-24.9 kg/m^2^), 75 points for overweight (25-29.9 kg/m^2^) or underweight (<18.5 kg/m^2^), 50 points for obesity class 1 (30-34.9 kg/m^2^), 25 points for obesity class 2 (35-39.9 kg/m^2^), or 0 points for extreme obesity class 3 (≥40 kg/m^2^) [72].

Finally, the social network score (Wakanet) is calculated based on the user number of contacts and the mean Wakastatus values of them.

Moreover, Wakamola is a multilanguage chatbot, including Spanish, English, and Catalan, allowing other languages to be easily included to the chatbot by adding corresponding dialogue file translation. The Wakamola chatbot is available in open access [73] under a Creative Commons license.

Focusing on the technical implementation, the chatbot engine of Wakamola is implemented as a Telegram bot using Python 3 [34]. Further technical details can be found in Multimedia Appendix 1.

### Usability Evaluation

As part of the chatbot’s user-centered development, a usability evaluation was carried out. The usability test focused on the process and the information user’s understanding. The usability test was designed as a face-to-face, assisted session. As a requirement to perform the test, it was mandatory to have a smartphone with Telegram installed on it. First, to characterize the sample, participants answered questions about gender, age, Telegram experience, messaging system used, and previous knowledge and experience regarding bots. Participants were then asked to perform a set of 6 specific tasks with the chatbot. Finally, the participants in the study responded to the System Usability Scale (SUS) questionnaire [36]. Further details about the usability evaluation can be found in Multimedia Appendix 1.

### Pilot Study

To test the feasibility of the chatbot, we conducted a pilot study with 85 university students (volunteers) recruited face to face. Participants were asked to complete all of the chatbot’s questions from the Personal, Diet, and Activity sections and to share the chatbot between them to build the social network. From the collected data, we obtained basic statistics from sociodemographic data, Wakamola scores, and BMI. Finally, we developed a free-access online tool [74] to perform the social network analysis by visualizing the network. The network visualization highlighted the users’ BMI and Wakastatus and showed communities obtained based on Louvain algorithm [75].

## Results

In this section, we show results from the users’ needs research survey and expert panel and from the usability test. We then show outcomes from the pilot study.

### Users’ Needs Investigation Results

Participants in the survey were recruited by email invitation from the Vice-Rector for Social Responsibility to the UPV’s university community (students, academy, and staff). The invitation included a brief description of the study and a link to the questionnaire. All participants who completed the questionnaire were included in the study. In total, 452 adults (197 males, 43.6%, and 255 females, 56.4%) participated in the survey for 11 days (Tables S3 and S4 in Multimedia Appendix 1). The sample was representative of the male and female composition of the university community and of a wide age range (from 18 to older than 65 years); likewise, it includes people from different lifestyles.

A high number of participants thought they were overweight (176/452, 38.9%). The perception of overweight increased with age. Most of them indicated having healthy dietary habits, including more women than men, at all ages. However, only half of the participants had regular physical activity. Moreover, almost half of them (217/452, 48.0%) thought that with their current habits, they might have problems of overweight in the future; this was seen more frequently in women than in men. Young adults had the highest percentage of self-perception of future overweight with current habits for both men and women. Most of the participants (325/452, 71.9%) would use the chatbot for obesity risk assessment and recommended it (406/452, 89.8%), mostly by talking about it, followed by through the medical centers and in their social networks. In addition, most participants believed that it would help to prevent obesity. They would prefer functionality regarding physical activity and diet recommendations, as well as about obesity risk assessment. Participants preferred colors in the field of obesity and overweight were, in order from highest to lowest, green, blue, and white. Participant’s graphical preferences were based on colors, simplicity, and figures. As well, quite a few of them would like a character associated with the app (“Wireframes results” and Figure S2 in Multimedia Appendix 1).

From the expert panel interviews, we identified the personal, diet, and physical activity questions, as well as the status assessment method (Wakastatus), already described in the chatbot’s design and functionality section.

### Usability Test Results

Participants were volunteer students recruited face to face in the Design School. In total, 61 students (young adults, mean age 20.5 years) participated in the usability test. All participants used a smartphone with Telegram previously installed on it. All participants were able to start Wakamola in Telegram without help, although most of them were not regular users of this messaging system. As a result, most users, when asked, would prefer that Wakamola be a separate app that could be installed on their mobile phone without Telegram. Most participants were able to understand all questions in the Personal, Diet, and Activity sections; however, they considered the Diet section to contain too many questions (23/61, 38%), while the number of questions was acceptable in other sections.

According to the SUS questionnaire [36], about half of the participants indicated acceptable usability. Further information about usability results is in Multimedia Appendix 1.

### Pilot Study Results

We carried out a pilot study with 85 university students recruited face to face. We filtered participants that completed all sections, 74 people in total (54 female, 20 male), for the data analysis. The mean age was 20.7 years, and the mean weight was 62.65 kg (SD 10.21). There were no participants with obesity-related diseases such as hypertension, diabetes, high cholesterol, or cardiovascular disease. The participants were from 55 different living areas according to their zip codes, most of them near the university area.

The percentage of people with overweight was 6.8% (5/74 people), while the percentage of people with underweight was higher at 10.8% (8/74 people). No obesity cases were detected in the sample.

The mean BMI was 21.4 (SD 2.41), which corresponds with normal weight. The mean Wakastatus was 78.3 (SD 10.67) on a scale of 1 to 100, mean Diet score was 63.6 (SD 4.67), mean Activity score was 65.3 (SD 32.91), and mean social network score was 26.6 (SD 13.12).

The most consumed types of food were olive oil, milk and derivatives, cereals, vegetables, and fruits. The less consumed types of food were seafood, butter, French fries, and sweetmeats. The consumption of alcohol and soft drinks was also low (Table 2).

Participants practiced physical activity regularly during the week. They spent a mean of 30.57 hours per week sitting and 7.02 hours per day resting. Table 3 shows the sample physical activity, sitting, and sleep hour habits in a week.

**Table 2:** Types of foods consumed weekly.

Food type	Units per week, mean
Seafood	0.54
Soft drinks with sugar	0.62
Butter	0.67
Alcohol drinks	0.78
French fries	1.18
Sweetmeats	1.22
Blue fish	1.64
Rice	2.04
Legumes	2.04
White fish	2.07
Sausage	2.13
Meats	2.27
Other oils	2.36
Cheeses	2.43
Nuts	2.65
Fruits	2.83
Vegetables	3.17
Cereals and derivatives	3.17
Milk and derivatives	4.43
Olive oil	12.72

**Table 3:** Physical activity and sleep hours in mean values per week.

Activity	Values, mean
Vigorous physical activities (times per week)	2.34
Vigorous physical activities (minutes)	33.97
Moderate physical activities (times per week)	5.11
Moderate physical activities (minutes)	35.76
Walked at least 10 continuous minutes (days per week)	5.80
Walking time (minutes)	34.26
Sitting (hours per week)	30.57
Sleep (hours per day)	7.02

**Figure 2:**
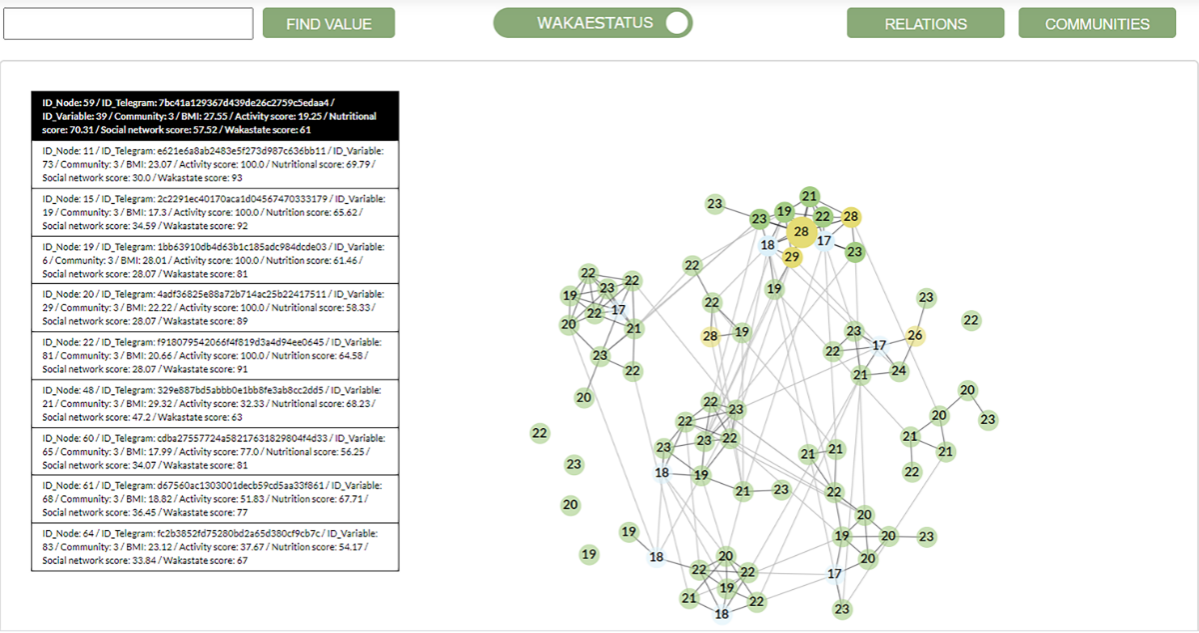
Target population network and communities representation, with nodes of the same community linked with dark gray edges and a user selected. BMI is shown inside nodes, and colors are based on BMI: blue (underweight, <18.5), green (normal weight, 18.50-24.9), yellow (overweight, ≥25), red (obesity, ≥30). BMI: body mass index.

We applied the online tool [74] to interpret collected data and showed it as a network graph with 74 users and 178 relations, including 5 users without relations and 12 social groups (communities) (Figure 2). Focusing on the 8 communities with more than one member, 3 of them (38%) had members with overweight, and 6 had members with underweight (75%). All individuals without connections were normal weight. The biggest community had 12 members; it was also the community with the highest percentage of overweight members, with 3 cases (25%).

Figure 2 shows users as nodes colored and labeled according to their BMI value: blue (underweight, <18.5), green (normal weight, 18.50-24.9), yellow (overweight, ≥25), or red (obesity, ≥30). The Wakastatus option allows it to be shown in the nodes. In Figure 2, a user has been selected and highlighted in a community; a table shows the BMIs and scores of his or her relations and contacts.

## Discussion

### Summary of Findings

We have translated standard questionnaires, traditionally used to collect data about sociodemographics, diet, and physical activity, to a novelty Telegram’s chatbot [73]. As well, we have defined a new user-friendly score to assess the user’s obesity risk based on his or her diet, physical activity, BMI, and social contacts’ lifestyles. Gamification principles have guided the design of the chatbot to help create a positive user experience. Moreover, we have confirmed that people are concerned about their weight and that they consider mHealth apps to be likely to help obesity prevention, as they are interested in using them. In a pilot study deployed in the academic community, we have been able to create a social network to study social factors influencing obesity. The researchers can access an online tool to graphically show the social network to aid data interpretation.

### Survey Findings

From the survey about users’ needs, we realized a need regarding overweight and obesity apps. This result could be linked with registered participants’ worry about their overweight, as 176 out of 452 (38.9%) indicated self-perception of being overweight, and 217 (48.0%) indicated that they could become overweight in the future with their current habits. Moreover, 325 out of 452 (71.9%) participants would use an app to know their obesity risk. As well, 406 out of 452 (89.8%) of them would recommend it. Furthermore, the number of survey responders (452 people) could be an indicator reflecting the concern about overweight and obesity in the university community involved in this study.

Weight management apps represent a popular area of mHealth today [37]. However, there is a need for trustable overweight and obesity apps; most of the commercial mobile apps for weight loss and management lack important evidence-based features, do not involve health care experts in their development process, and have not undergone rigorous scientific testing [38]. Wakamola’s chatbot could contribute to cover this need because it involves experts, is based on scientific evidence, and has been subjected to an exhaustive testing process.

Regarding the 52 wireframes scored, we finally selected one based on a character (Wakamola). This selection allowed us to implement personification, the attribution of a personal nature or human characteristics [76] to the chatbot. Personification has a positive effect on the user experience [39]. The introduction of humanlike cues in a chatbot increase users’ emotional connection [18]. As well, previous studies suggest a significant effect of anthropomorphic design features (human characteristics) on perceived usefulness, with a strength 4 times the size of the effect of functional chatbot features [40].

### The Chatbot as a Feasible Tool to Collect Data

We here propose a chatbot as a novel tool to collect data associated with overweight and obesity. Chatbots could help to collect data in a longitudinal and long-term way [8] that would be difficult and time-consuming with traditional methods, such as standardized questionnaires [77]. Several studies state that a feature that can engage users in completing questionnaires is their presentation through a chatbot [8,18]. Users are more likely to answer questions through a chatbot than in a questionnaire or interview because they connect them with entertainment and novelty, and they are curious about them [19]. Moreover, there is previous research about the application of chatbots to collect data associated with obesity and overweight [10-13]. Furthermore, the use of chatbots has also extended to other health fields such as oncology [78].

We collected data in a pilot study with 85 people. Analyzing the data obtained from the pilot, we found a percentage of people with overweight of 7% (5/74), while the percentage of people with underweight was higher (8/74, 11%); no obesity cases were detected in the sample. The presence of underweight cases could be explained by the higher representation of women in the sample (54/74, 73%), previous studies indicates that women were more likely to be underweight than men [79].

The most consumed types of food were olive oil, milk and derivatives, cereals, vegetables, and fruits, all of which are types of foods associated with the Mediterranean diet [41]. However, other dietary habits with restricted consumption in the Mediterranean Pyramid [41], such as consumption of butter, French fries, sweetmeats, alcohol, and soft drinks, were low [41]. Moreover, most of the participants practiced regular physical activity (Table 1) and slept for a mean of 7 hours, which is a good rest time in adults [80]. These results could explain the participants’ mean BMI of 21.4 (SD 2.41), which corresponds to normal weight.

We applied the developed online tool [74] to interpret collected data and showed it as a network graph with 74 nodes and 178 relations, including 5 nodes without relations, and 12 communities based on the Louvain algorithm [75] (Figure 2). The biggest community had 12 members; it was also the community with the highest percentage of overweight members (3 cases out of 74, 25%). Further research would need to study if there is an overweight “contagion” effect in this community [5] or if individuals with similar BMIs are clustering together into this group [6].

This approach and further development of the tool would support the study of overweight and obesity causes, not only from the point of view of the habits of people, but also from the perspective of the influence of their relationships and socioeconomic environment. We recall that previous studies have used social network analysis to study the overweight and obesity problem [81,82] (Multimedia Appendix 1, “Social networks influence in the development of Obesity and Overweight”). It should be noted that the relationships created in the chatbot also specify if it is a relation with a person from home, a family member, a friend, or a coworker, so further research should approach the influence of these subnetworks on the population under study regarding overweight and obesity.

### Chatbots to Assess Lifestyle

In Wakamola, diet is scored based on the type and frequency of foods, and physical activity habits are also scored; these are relevant parameters to control body weight [2]. The user’s weight and height are also collected to calculate their BMI, which is a widely applied fat mass indicator parameter. Users are informed about their BMI, which is a value unknown to most people, and warned if it is over the recommended values for a normal weight.

Moreover, users get a global score of their status according to input data (Wakastatus), although this score needs further study, for example, regarding the correlation of BMI with defined Wakastatus, diet, activity, and social scores, as well as with other overweight and obesity indicators.

### Lessons Learned About the Wakamola Chatbot Design

People are curious about what chatbots are and how they work as a recent technology, which is reflected by the interest in Wakamola in the media after its launch [83]. Based on our experience, people are more likely to use a chatbot in a messaging platform they already use than to install another app in their phones or visit a website. However, after the first approach to the chatbot, people expect more feedback to engage the app. The obesity risk assessment alone is not enough; people ask for recommendations about diet and physical activity (general and personalized), tracking (diet, physical activity, calories consumed), community, sharing progress, positive messages, information about nutrition and healthy habits, success stories, syncing with activity bracelets, and rewards for improving, among others (Table S4 in Multimedia Appendix 1).

The use of a character with personalization helps users to empathize with it and promote the app’s use. Based on our experience with Wakamola so far, we know that people want to meet Wakamola after seeing the character image. However, after starting the chatbot, people expect more feedback to become regular users; most of the users use it one time. Thus, the chatbot needs additional effort to improve engagement to enable long-term control studies.

The usability and acceptance problems detected were mainly related to the dependency on the Telegram platform (“Usability test results” in Multimedia Appendix 1). Participants that were not Telegram users before using Wakamola needed help to share it. Moreover, they were unlikely to use it by their own initiative. The decision to develop the chatbot in a third-party platform has advantages, such as speeding up the development and removing the requirement for regular users to install a new app to use the chatbot, saving storage space in their phones. However, this dependency reduces the acceptance of the app for people unfamiliar with the platform because they think that the effort required is higher than installing only an independent app. Moreover, this dependency slows down the expansion of the app and therefore the creation of the network to support the study of social causes of obesity. Thus, we consider that the chatbot Wakamola needs to be multiplatform and an independent online chatbot to reach the maximum number of potential users. As well, the sharing procedure requires an in-depth study to achieve stickiness and usability. Furthermore, the perception of trust is fundamental for acceptance and to extend the use of the chatbot. People would open an invitation to a chatbot only if it includes information about the app objectives and comes from a reliable source.

Moreover, the number of chatbot messages needs to be limited to avoid user fatigue and abandonment. Thus, the Wakamola Diet section in particular needs to be shortened.

From the data collection perspective, the Wakamola chatbot enables the definition of different instances, which could be useful to perform parallel pilot studies in target populations. Two new pilot studies are in process, involving 1500 people so far [84,85].

### Conclusions

The Wakamola chatbot provides a tool to collect linked data about users’ sociodemographics, overweight- and obesity-related diseases, diet and physical activity habits, BMI, social network, and environment. All these data could aid the study of overweight and obesity in a target population. Moreover, the social network created with the chatbot allows the study of overweight and obesity from a social approach; an online tool has been developed to support it. As well, the chatbot is an end user tool for self-assessment of overweight and obesity risk. Results indicate that this new chatbot meets the needs of both end users and experts, although usability and feedback should keep improving. Moreover, its user-centered design would contribute to the chatbot’s usability and acceptance in real scenarios.

However, we are aware of the limitations of this preliminary study. The cohort in the pilot study might not be representative due to selection bias. We plan to apply Wakamola in wide populations in a real context to analyze the data and social network. Moreover, we intend to study the feasibility of the chatbot to help overweight and obesity screening and interventions.

Further studies will focus on Wakamola’s usability improvement, collecting data in large populations for social network analysis, the chatbot’s messaging multiplatform compatibility, the study of gamification perception and its effects on the user, and chatbots’ performance in comparison to traditional graphical user interfaces in applications in the field of obesity and overweight.

In addition, a Wakastatus score validation is required to clarify its perception by users and its feasibility to assess users’ obesity risks.

## Appendix

Multimedia Appendix 1 Wakamola supplementary material.

Multimedia Appendix 2 Wakamola chatbot.

## Abbreviations

IPAQ: International Physical Activity Questionnaire
mHealth: mobile health
SUS: System Usability Scale
UPV: Universitat Politècnica de València
