# Supplementary material for: A User-Centered Chatbot (Wakamola) to Collect Linked Data in Population Networks to Support Studies of Overweight and Obesity Causes: Design and Pilot Study
Source: JMIR Med Inform. 2021 Apr 14;9(4):e17503. doi: 10.2196/17503 (PMC8087340; doi:10.2196/17503)
Supplement: Multimedia Appendix 1 [file medinform_v9i4e17503_app1.docx]

### Multimedia Appendix 1. Wakamola supplementary material.

### Introduction

#### **Social networks influence in the development of Obesity and Overweight**

The term *social network* defines a set of individuals and the ties among them [1]. Social network analysis refers to personal networks or ego networks (egocentric networks), with an individual at the center, or to the whole or complete networks (sociometric networks), with entire networks of networks at the level of communities [2].

The effect of social networks on health is a topic of growing interest, particularly in an increasingly connected world [3]. Fowler and Christakis in their study about the spread of obesity in a large social network over 32 years [4] found evidence of the "contagion" of obesity among people in close social circles, indeed the relevant finding in the Framingham heart study suggests that ties between friends have even more significant effect on a person's risk of obesity than genes. A person's chances of becoming obese increased by 57% if he or she had a friend who became obese in a given interval. Moreover, for a wide variety of conditions and networks, Bahr *et al.* showed that individuals with similar Body Mass Indexes would cluster together into groups [5].

A scoping review among forty five papers [6] identified three inter-related social processes that explained how social networks might influence the development of overweight and obesity: *social contagion* (whereby the network in which people are embedded influences their weight or weight-influencing behaviors over time), *social capital* (whereby sense of belonging and social support influence weight or weight-influencing behaviors), and *social selection* (whereby a person's network might be developed according to his or her weight). Moreover, this review identified six different types of networks that affect overweight and obesity: a paired network (one's spouse or intimate partner); friends and family (including work colleagues and people within social clubs); ephemeral networks in shared public spaces (such as fellow shoppers in a supermarket or diners in a restaurant); people living within the same geographical region; peers (including co-workers, fellow students, fellow participants in a weight loss program); and cultural groups (often related to ethnicity). This study revealed that processes of contagion were most common within friends and family networks, peer networks and cultural groups, supporting Fowler and Christakis 's findings [4].

In our study we propose a Chatbot as an mHealth tool to collect individuals’ data about lifestyle and socio-demographics, and also to connect them building a social network that would support studies to understand how social processes influence the development of overweight and obesity.

#### **User-Centered Design in mHealth**

Numerous mHealth apps being developed under a User-Centered Design (UCD) approach [7–10], some of them focus on obesity [11–16]. UCD is a collaborative, evidence-based approach that incorporates the needs and context of a specific end-user group to inform the development and design of mHealth apps [10].

The UCD approach in mHealth mostly includes user’s needs investigation, prototype development, and usability evaluation [17]. To perform UCD multiple strategies can be used, such as card sorting, contextual inquiry, focus groups, interviews, log file analysis, paper prototyping, surveys, task analysis, usability test, expert review, guided walkthrough, heuristic evaluation, *personas*, etc. [18]. For healthcare apps, interviews with subject matter experts and other [relevant stakeholders](https://www.sciencedirect.com/topics/computer-science/relevant-stakeholder) contribute to a better understanding of products' opportunities and challenges [19].

Jefferson *et al.* applied UCD to engage mothers in the development of an mHealth breastfeeding application [20]; they used a survey, focus groups and System Usability Scale (SUS) test [21]. Birnie *et al.* [10] developed an app for self-management of postoperative pain in children and adolescents and involved children, adolescents, and parents to complete individual semi-structured interviews, and health care providers participated in focus groups. Zhou et al. [22] applied UCD to building a mobile Personal Health Record app. A large-scale questionnaire study was conducted with the general population to gain an understanding of their needs and expectations. Then a usability study was performed with 15 participants who were asking to finish a set of tasks and to respond to a usability test (Post-Study System Usability Questionnaire (PSSUQ) [23]). Pricilla et al. [24] designed a Chatbot-Based Conversational Commerce with UCD applying users’ needs survey and interviews, and usability testing for evaluating the prototype including interviews, task testing and questionnaires. Vilardaga et al. [25] designed a smoking cessation smartphone app for people with serious mental illness, and they used UCD methods such as expert panel guidance, the creation of *personas* [26], sketching and paper prototyping, and usability testing. To simulate the app experience, they used prototyping software. Usability testing procedures consisted in completing a series of tasks with the simulated app, evaluation of user experience with semi-structured interviews, and (3) rating the prototype using the system usability scale (SUS) [21].

When patients are involved in the design of a healthcare system, the term Patient-Centered Design (PCD) emerge [27–29]. Patient-Centered Design (PCD) is a particular UCD where the end-user is a patient who will use the solution for healthcare [30].

According to previous studies, to design our Chatbot under a UCD approach, we decided to apply: users open survey, interviews with users and experts, sketching (50 wireframes), prototyping, set of tasks test, and the System Usability Scale questionnaire (SUS) [21].

### Wakamola chatbot´s technical development

The Chatbot engine of Wakamola is implemented as Telegram bot using Python 3[40]. It is a finite-state machine in which information is kept in the database, and its initial state runs to the main menu. When a questionnaire is selected, the Chatbot asks the first question and updates the state machine. Upon receiving the response from the user, it is checked for correctness: format and range of accepted values. If the answer is correct, the Chatbot saves it in the database, advances the state machine, and emits the following question. If the answer is incorrect, the Chatbot engine repeats the question.

When a user completes all the questions of a questionnaire, the Chatbot comes back to the initial state, and it shows the user the main menu again. Users can jump between options using the specific command (usually through a button) but will always point to the first question, although the previous answers are stored in the BD.

The finite-state machine for each user is maintained in a MariaDB database (https://mariadb.org/). The same database stores the answers provided by users. All answers are kept anonymous. To achieve this, we use MD5 hash function [41] to transforms the uncorrelated Telegram user number assigned by the platform into a 128-bit alphanumeric string that also has no reverse transformation.

Figure S1 shows the process that follows the communication: when a user sends a message to the Chatbot, it sends a message to the Telegram servers, where this message is registered. On its behalf, the Chatbot makes periodic requests to these servers, through Telegram's public API to retrieve the new messages. The relevant information from each message is collected, the necessary action is taken, and the state of the finite-state machine is updated. If this action includes responses to the user, the Chatbot responds using the Telegram's public API.


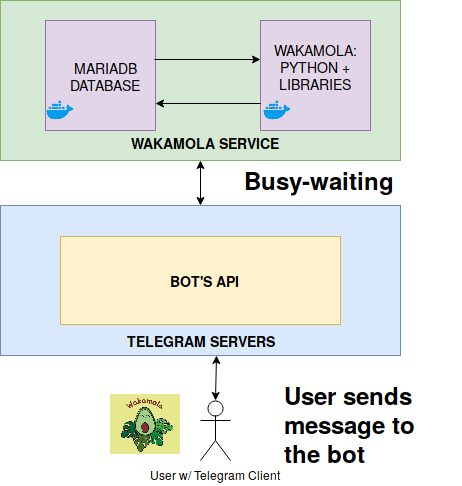


Figure S1. The system architecture of the Chatbot Wakamola.

### Wakastatus score definition

Table S1 shows the criteria for scoring each group of food based on their frequency of consumption according to [42]; groups 10 and 11 in Table S1 are scored based on Mediterranean Diet Pyramid [43]. Table S2 shows the diet items and corresponding group of food for scoring. So that, each item (food) is scored according to its group of food (Table S2), and its frequency of consumption (Table S1), then all item´s scores are summarized and normalized in a 1 to 100 scale to calculate a diet score. Figure S2 shows an example of the diet´s questions in the Chatbot.

Table S1. Criteria for a scoring group of foods based on their frequency of consumption (adapted from [42])

| **Nº** | **Group of food** | **Criteria for maximum score=10** | **Criteria for score=7,5** | **Criteria for score=5** | **Criteria for score=2,5** | **Criteria for minimum score=0** |
| --- | --- | --- | --- | --- | --- | --- |
| **1** | Cereals and derivatives | Daily consumption | 3 or more times a week | 1 or 2 times a week | Less than once a week | Never or almost never |
| **2** | Vegetables | Daily consumption | 3 or more times a week | 1 or 2 times a week | Less than once a week | Never or almost never |
| **3** | Fruits | Daily consumption | 3 or more times a week | 1 or 2 times a week | Less than once a week | Never or almost never |
| **4** | Milk and derivatives | Daily consumption | 3 or more times a week | 1 or 2 times a week | Less than once a week | Never or almost never |
| **5** | Meats | 1 or 2 times a week | 3 or more times a week | Less than once a week | Daily consumption | Never or almost never |
| **6** | Legumes | 1 or 2 times a week | 3 or more times a week | Less than once a week | Daily consumption | Never or almost never |
| **7** | Sausage/Soft drinks without sugar | Never or almost never | Less than once a week | 1 or 2 times a week | 3 or more times a week | Daily consumption |
| **8** | Sweetmeats | Never or almost never | Less than once a week | 1 or 2 times a week | 3 or more times a week | Daily consumption |
| **9** | Soft drinks with sugar | Never or almost never | Less than once a week | 1 or 2 times a week | 3 or more times a week | Daily consumption |
| **10** | Olive oil; Other oils: sunflower, soybean, etc.; Butter | 3 times/day | 4 times/day | 5 times/day | 6 times/day | >6 times/day |
| **11** | Nuts | 3-7 times/week | 8 times/ week | 9 times/ week | 10 times/ week | >10 times/ week |

Table S2. Classification of foods by group of food.

| **Foods** | **Group of food** |
| --- | --- |
| Breakfast cereals; Bread in a sandwich or with meals; Wholemeal bread. Potatoes (not fried).  Noodle or rice soups; White rice, paella, Pasta: noodles, macaroni, spaghetti, | 1 (Cereals and derivatives) |
| Salad: lettuce, tomato, endive, gazpacho; Green beans, chard or spinach.  Garnish vegetables such as eggplant, mushrooms or vegetable creams | 2 (Vegetables) |
| Citrus fruit: orange, mandarin; Other fruits: apple, pear, peach, banana…  Natural and commercial fruit juices; | 3 (Fruits) |
| Milk, yoghurt.  Cooked ham type York; White or fresh cheese or low in calories | 4 (Milk and derivatives) |
| Eggs; Chicken or turkey; pork, lamb, steak; Whitefish: hake, grouper; Bluefish: Sardines, tuna, salmon; Seafood: mussels, prawns, prawns, squid; Other cheeses: cured, semi-cured, creamy | 5 (Meats) |
| Legume: lentils, chickpeas, beans, soybeans | 6 (Legumes) |
| French fries; Minced meat, sausage, hamburger, veal, sausages; Serrano ham, cold meat.  Beer without alcohol; Low-calorie drinks Coca-Cola Light or Zero. | 7 Sausage/Soft drinks without sugar |
| Biscuits without filling or covering. Chocolate: bar, chocolates, bars or others; Biscuits with chocolate or cream, with filling; Cupcakes, sponge cake; Ensaimada, donut, croissant ; Canned or syrupy fruit; Croquettes, dumplings, pizza; Dairy desserts: custard, flan, cottage cheese; Cream or chocolate cakes; Bags of snacks type (snack) chips…; Confectionery: gummy jellies, candy …; Ice creams | 8 (Sweetmeats) |
| Sugared drinks such as Coca-Cola, Fanta or other soft drinks. | 9 (Soft drinks with sugar) |
| Olive oil; Other oils: sunflower, soybean, etc.; Butter | 10 (oils and butter) |
| Nuts: peanuts, hazelnuts, almonds... | 11 (Nuts) |

Equation 1 shows how to calculate the social net Score (Wakanet).

SSu=min((max(WSCu− −WSu,0)MAXnetwork)+log2|WSCu|,MAXnetwork),  Eq. (1)

*Where:*

*SS_u_: Social score of user u*

*WS_u_: wakastatus of user u*

*WSC_u_: wakastatus of user u*

*MAXnetwork* *: a constant to limit the weight of the Social score in the final Wakaestatus score, default value = 10.*

#### **Gamification implementation in the chatbot**

The Wakamola Chatbot implements gamification strategies such as a system of points (scores), feedback (Wakastatus and social network status and BMI), and emotional engagement with a character to provide gameful experience to the user and increase his/her adherence to the app, also trying to break with the overweight and obesity stigma. We also included a user’s status score not only to promote sharing the Chatbot but also as a reward to reflect users compromise with the promotion of healthy habits in food and physical activity in their social environment. Moreover, we have included the use of emoji added to the Wakamola´s text-messages that would contribute to a more realistic and friendly conversation [44]. However, recent research suggests that the implementation of gamification is highly context-dependent [45] and that it might as well lead to negative effects [46]. In a context like obesity and overweight, implementing gamification elements like points or leaderboards in conjunction with social features could lead to unintended effects. Thus, the Wakamola gamification needs further research to clarify its positive effect on the users.

### User´s need Survey

#### **Sample**

Table S3. Characteristics of the sample.

| **Characteristic** | **Values #(%)** |
| --- | --- |
| Number of participants | 452 (100%) |
| **Gender** |  |
| Female | 198 (56,19%) |
| Males | 254 (43,81%) |
| **Age (years)** |  |
| 18-25 | 166 (36,73%) |
| 26-35 | 57 (12,61%) |
| 36-45 | 89 (19,69%) |
| 46-55 | 65 (14,38%) |
| 56-65 | 41 (9,07%) |
| > 65 | 34 (7,52%) |
| **Type of living** |  |
| Living alone | 55 (12,17%) |
| Student residence | 4 (0,88%) |
| Living with their families | 283 (62,61%) |
| Shared a flat | 68 (15,04%) |
| Others | 42 (9,29%) |
| **Overweight (self-perception**) | 177 (39,16%) |
| Female | 82 (46,33%) |
| Males | 95 (53,67%) |
| **Overweight (self-perception) by Age** |  |
| 18-25 | 42 (25,30%) |
| 26-35 | 19 (33,33%) |
| 36-45 | 38 (42,70%) |
| 46-55 | 33 (50,77%) |
| 56-65 | 24 (58,54%) |
| >65 | 21 (61,76%) |
| **Future overweight (self-perception)** | 221 (48,89%) |
| Female | 120 (54,30%) |
| Males | 101 (45,70%) |
| **Future overweight (self-perception) by age** |  |
| 18-25 | 67 (30,32%) |
| 26-35 | 26 (11,76%) |
| 36-45 | 49 (22,17%) |
| 46-55 | 34 (15,38%) |
| 56-65 | 25 (11,31%) |
| >65 | 20 (9,05%) |
| **Healthy diet perception** | 325 (71,90%) |
| Female | 192 (59,08%) |
| Males | 133 (40,92%) |
| **Healthy diet perception by age** |  |
| 18-25 | 108 (65,06%) |
| 26-35 | 42 (73,69%) |
| 36-45 | 65 (73,03%) |
| 46-55 | 51 (78,46%) |
| 56-65 | 29 (70,73%) |
| >65 | 30 (88,23%) |
| **Regular physical activity** | 243 (54,00%) |
| Female | 135 (55,56%) |
| Males | 108 (44,44%) |
| **Regular physical activity by age** |  |
| 18-25 | 76 (45,78%) |
| 26-35 | 33 (58,93%) |
| 36-45 | 47 (52,81%) |
| 46-55 | 42 (64,62%) |
| 56-65 | 26 (63,41%) |
| >65 | 19 (57,58%) |

### Results

#### **Survey results**

Table S4. Questions and answers about an obesity and overweight app users’ preference.

| **Questions** | **Positive answers # (%)** | |
| --- | --- | --- |
| Number of participants | 452 (100%) | |
| Do you think you would use an app to know your obesity risk? | 327 (72,35%) | |
| Would you recommend an app for obesity risk assessment? | 407 (90,04%) | |
| **How would you recommend an App for obesity risk assessment?** | | |
| Talking about her | 296 (65,49%) | |
| Through the medical centers | 194 (42,92%) | |
| From my social networks | 153 (33,85%) | |
| Sending invitation from the App itself | 103 (22,79%) | |
| With Google ads | 60 (13,27%) | |
| Through the digital press | 58 (12,83%) | |
| Other (specify) | 14 (3,10%) | |
| **What features would you like to have an app on obesity?** | | |
| Physical activity | 406 (89,82%) | |
| Diets | 392 (86,73%) | |
| Obesity risk assessment | 379 (83,85%) | |
| Recommend nutritionists | 301 (66,59%) | |
| Psychological support | 193 (42,70%) | |
| Other recommendations (free text) | 38 | Recommendations, progress tracking, gamification, pedometer, community, share progress, personalize diet, calories consuming, positive message, daily diet and physical activity tracking, information about nutrition and healthy habits, comics, photos album, success stories, dietary advice, sync with activity bracelets, recompense, reward for improving |
| Do you think that an App to identify the risk of obesity can help prevent this condition? | 312 (69,03%) | |

#### **Wireframes results**

Regarding to the wireframes, 52 were designed by 150 students from the School of Design of Universitat Politècnica de València and scored 1 to 5 in the survey. Participant’s main criteria to score the wireframes alternatives were colors, simplicity, and figures. We found the remarkable results that the 3rd preferred alternative (score by 3,1), quite close to the first alternative (score by 3,3) and the second one (score by 3,2), included a character named Wakamola as the image and name of the app (see Figure S2). We realized that this design would facilitate to implement chatbots’ personalization to achieve the positive effect of it on the user experience [47], to increase users´ emotional connection with the chatbot [48], and to improve perceived usefulness [49]. Thus, we decided to choose Wakamola´s option according to user´s preferences and the chatbot´s personalization potential.

Participants were asked about their color’s preferences for and app about O&O (see Figure S2). Preferred colors were, in order from highest to lowest: green, blue and white.


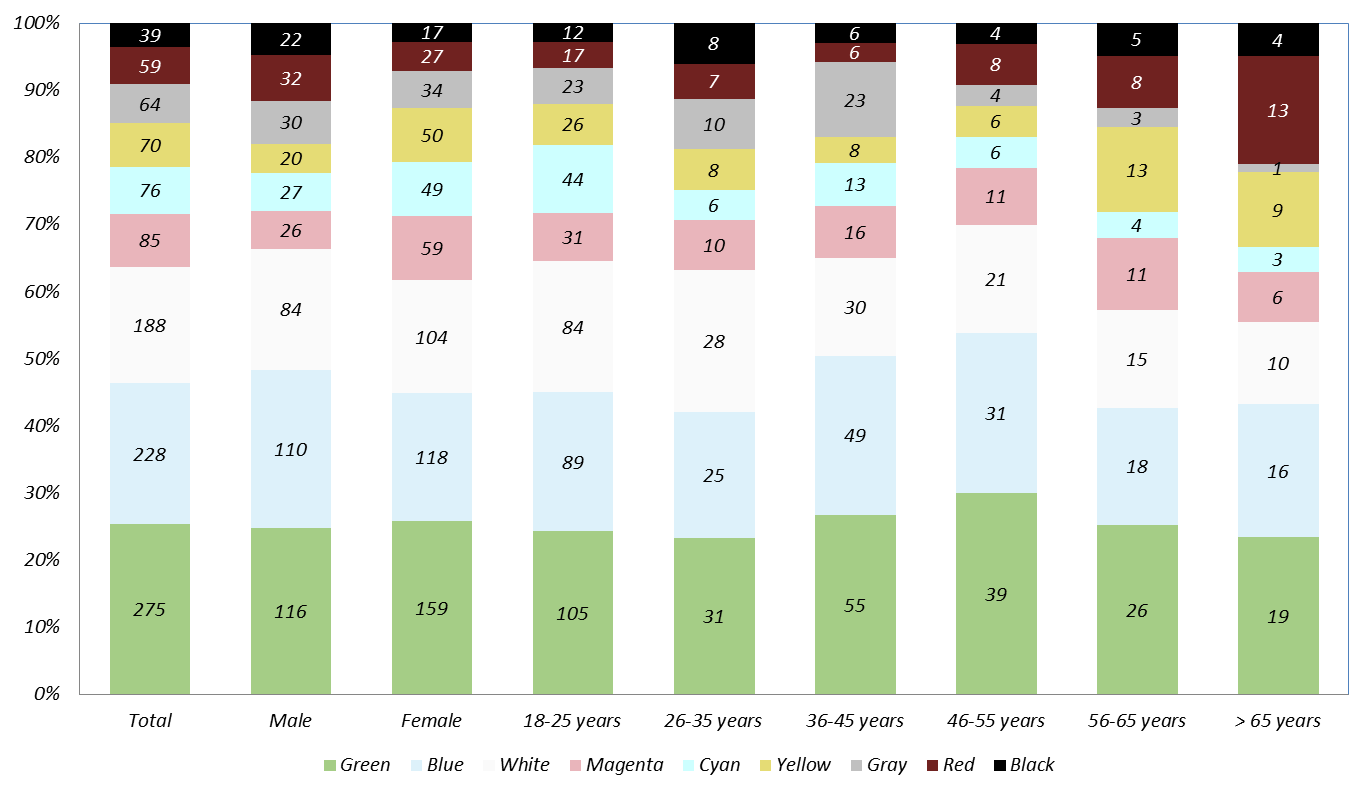


Figure S2: Participants colors' preferences for an O&O app (visualization in Wakamola’s colors palette).

#### **Chatbot appearance for acceptance: character, colors, and act on user’s feelings**

The Chatbot is based on the character Wakamola, previous studies suggest a significant effect of anthropomorphic design features on perceived usefulness, with a strength four times the size of the effect of functional Chatbot features [49]. Moreover, the use of emoji added to the text-messages would contribute to a more realistic and friendly conversation [44].

Our findings of the suitable colors for the design of an O&O app according to users’ preferences (green, blue, and white) are coherent with previous studies regarding the color and its effect on feelings. According to these studies, the blue color is the people´s preferred and is associated with sympathy, harmony, friendship, and trust [50]; serenity, security, and well-being [51]. Green is the second preferred color and is associated with life, health, natural, fresh, and hope [50]; joy, calm, energy, freedom, vitality, health, and balance [51]. The white color is associated with the beginning and the new, the good, the truth, the ideals, the perfection, the honesty, the univocal and the exact [50]; peace, cleanliness, transparency, neatness, purity, innocence, tranquility, serenity, calm, clarity, light, space, sincerity and goodness [51]. Green and blue are the colors most associated with pleasantness and tolerance [50]. The green, blue, and white combination is associated with tranquility. From app´s design point of view, we considered that especially the pleasantness and tolerance (green), truth and good (white), also health and balance (blue) perceptions could help to break with the stigma in an O&O app, also mentioned positive feeling could contribute to app´s user´s acceptance. So that, based on our survey results and the feeling color´s perception study, we have considered green, blue and white as main colors of the app’s design.

#### **Gamification implementation in Wakamola**

To implement gamification and personification in the Chatbot [49], taking into account the results of the survey, we decided to center the graphical design in character named Wakamola. The character was redesigned to achieve greater expressiveness and friendly face, mainly by adding eyes and mouth. Also, to simulate a more realistic and friendly conversation emoji were added according to the question’s content [44]. Stickers based on the character were prepared to be shown as first bot´s message in each app’s section according to users’ preferences in color and keyword features. Additionally, a Wakamola’s theme to personalize the background of the Chatbot based on the character and favorites colors resulted from the survey was designed.

### Usability evaluation

Participants were asked to perform a set of 6 specific tasks with the Chatbot: task 1: answer personal; task 2: answer diet; task 3: answer activity; task 4: consulting Wakastatus; task 5: consulting Wakanet; task 5: sharing Wakamola. After performing each task, participants provided the following measures: successful, time-consuming, number of errors, understanding, acceptable number of questions, agility in responses, and observation (free text). Finally, the participants in the study responded to the system usability scale questionnaire (SUS) [21]. SUS calculates a single score representing a measure of the overall usability of the system. The threshold of 51 points (50th percentile) represents an “Ok” level of perceived usability of the system scores, above 71 “Good” and above 85 “Excellent” [52].

#### **Usability test results**

Table S5. Wakamola’s pilot study results regarding bot’s and Telegram system knowledge.

| **Characteristic** | **Value #(%)** |
| --- | --- |
| Number of participants | 61 (100%) |
| Female | 48 (78,7%) |
| Male | 13 (21,3%) |
| Mean Age | 20,5 |
| **Operative system** |  |
| Android | 39 (64%) |
| iOS | 22 (36%) |
| **About Telegram** |  |
| Previous knowledge | 33 (54%) |
| Regular users | 4 (7%) |
| No answer | 24 (39%) |
| **About Chatbots** |  |
| To know what a Chatbot is | 36 (59%) |
| Previously used a Chatbot | 20 (33%) |
| Previously used a Telegram’s Chatbot | 4 (7%) |
| **Usual messaging system** |  |
| WhatsApp | 61(100%) |
| Telegram | 3 (5%) |
| Instagram | 6 (10%) |
| Outlook | 1 (2%) |
| Skype | 1 (2%) |
| Discord | 1 (2%) |
| Users would prefer that Wakamola as Telegram’s independent app | 40 (65,5%) |

In Wakastatus section, users indicated difficulty in understanding the information showed (23%). In Wakanet section, we detected problems in comprehension regarding sharing Wakamola process, 33% of participants needed help to be able to share Wakamola. Moreover, the help image message included in the bot was useful only for 57% of them.

In the free text observations users reported: "lack of feedback about if the sharing process has been successful"; "instructions are not clear"; "don´t able to share"; "don´t understand process or instructions"; "press and open the link in the same Chatbot"; "too difficult to share"; "too many messages at time". In the Wakanet section, the level of understanding of the information displayed was good.

In “Consulting Wakamola net task” most of the participants completed successfully but, 62% of them reported difficulties to understand the information.

Finally, about the Wakamola invitation message, some users would open it directly (22%); however, 33% would ask before opening it; moreover, 20% wouldn’t open it. When asked if the invitation was clear, 70% agreed, and 72% reported that it seems trustworthy. In the free text observation questions, some users indicated that more information about the app objectives would help to accept the invitation.

To complete the usability study of the Wakamola Chatbot, the participants responded to the System Usability Scale Questionnaire (SUS) [21]. Only 46% of participant´s SUS score was 51 or higher, corresponding to an “ok” level of perceived usability [52].

### References

[1] S. Wasserman, K. Faust, others, Social network analysis: Methods and applications, Cambridge university press, 1994.

[2] S. Nam, N. Redeker, R. Whittemore, Social networks and future direction for obesity research: A scoping review, Nursing Outlook. 63 (2015) 299–317. https://doi.org/10.1016/j.outlook.2014.11.001.

[3] M.C. Pachucki, E. Goodman, Social Relationships and Obesity: Benefits of Incorporating a Lifecourse Perspective., Current Obesity Reports. 4 (2015) 217–223. https://doi.org/10.1007/s13679-015-0145-z.

[4] J.H. Fowler, N. Christakis, The Spread of Obesity in a Large Social Network Over 32 Years., New England Journal of Medicine. 357 (2007).

[5] D.B. Bahr, R.C. Browning, H.R. Wyatt, J.O. Hill, Exploiting social networks to mitigate the obesity epidemic., Obesity (Silver Spring, Md.). 17 (2009) 723–728. https://doi.org/10.1038/oby.2008.615.

[6] K. Powell, J. Wilcox, A. Clonan, P. Bissell, L. Preston, M. Peacock, M. Holdsworth, The role of social networks in the development of overweight and obesity among adults: a scoping review., BMC Public Health. 15 (2015) 996. https://doi.org/10.1186/s12889-015-2314-0.

[7] M. Georgsson, N. Staggers, E. Årsand, A. Kushniruk, Employing a user-centered cognitive walkthrough to evaluate a mHealth diabetes self-management application: A case study and beginning method validation, Journal of Biomedical Informatics. 91 (2019) 103110. https://doi.org/https://doi.org/10.1016/j.jbi.2019.103110.

[8] M. Marko-Holguin, S.L. Cordel, B.W. Van Voorhees, J. Fogel, E. Sykes, M. Fitzgibbon, A.E. Glassgow, A Two-Way Interactive Text Messaging Application for Low-Income Patients with Chronic Medical Conditions: Design-Thinking Development Approach, JMIR MHealth and UHealth. 7 (2019) e11833–e11833. https://doi.org/10.2196/11833.

[9] C. Steele Gray, A. Khan, I. McKillop, S. Sharpe, C. Cott, User-centred co-design with multiple user groups: The case of the electronic Patient Reported Outcome (ePRO) mobile application and portal, International Journal of Integrated Care. 19 (2019) 439. https://doi.org/10.5334/ijic.s3439.

[10] K.A. Birnie, F. Campbell, C. Nguyen, C. Lalloo, A. Tsimicalis, C. Matava, J. Cafazzo, J. Stinson, iCanCope PostOp: User-Centered Design of a Smartphone-Based App for Self-Management of Postoperative Pain in Children and Adolescents., JMIR Formative Research. 3 (2019) e12028. https://doi.org/10.2196/12028.

[11] C.C. Tsai, G. Lee, F. Raab, G.J. Norman, T. Sohn, W.G. Griswold, K. Patrick, Usability and Feasibility of PmEB: A Mobile Phone Application for Monitoring Real Time Caloric Balance, Mobile Networks and Applications. 12 (2007) 173–184. https://doi.org/10.1007/s11036-007-0014-4.

[12] K.E. Curtis, S. Lahiri, K.E. Brown, Targeting Parents for Childhood Weight Management: Development of a Theory-Driven and User-Centered Healthy Eating App, JMIR MHealth UHealth. 3 (2015) e69. https://doi.org/10.2196/mhealth.3857.

[13] D. Fedele, R. Lucero, D. Janicke, M. Abu-Hasan, E. McQuaid, J. Moon, A. Fidler, T. Wallace-Farquharson, D. Lindberg, Protocol for the Development of a Behavioral Family Lifestyle Intervention Supported by Mobile Health to Improve Weight Self-Management in Children With Asthma and Obesity, JMIR Res Protoc. 8 (2019) e13549. https://doi.org/10.2196/13549.

[14] M. Bardus, A. Ali, F. Demachkieh, G. Hamadeh, Assessing the Quality of Mobile Phone Apps for Weight Management: User-Centered Study With Employees From a Lebanese University, JMIR Mhealth Uhealth. 7 (2019) e9836. https://doi.org/10.2196/mhealth.9836.

[15] S. Gabrielli, M. Dianti, R. Maimone, M. Betta, L. Filippi, M. Ghezzi, S. Forti, Design of a Mobile App for Nutrition Education (TreC-LifeStyle) and Formative Evaluation With Families of Overweight Children, JMIR MHealth and UHealth. 5 (2017) e48–e48. https://doi.org/10.2196/mhealth.7080.

[16] J. Rivera, A.C. McPherson, J. Hamilton, C. Birken, M. Coons, M. Peters, S. Iyer, T. George, C. Nguyen, J. Stinson, User-Centered Design of a Mobile App for Weight and Health Management in Adolescents With Complex Health Needs: Qualitative Study, JMIR Formativ Res. 2 (2018) e7. https://doi.org/10.2196/formative.8248.

[17] L. Griffin, D. Lee, A. Jaisle, P. Carek, T. George, E. Laber, B. Lok, F. Modave, E. Paskett, J. Krieger, Creating an mHealth App for Colorectal Cancer Screening: User-Centered Design Approach., JMIR Human Factors. 6 (2019) e12700. https://doi.org/10.2196/12700.

[18] A. Dopp, K. Parisi, S. Munson, A. Lyon, A glossary of user-centered design strategies for implementation experts, Translational Behavioral Medicine. (2018). https://doi.org/10.1093/tbm/iby119.

[19] T.B. Wray, C.W. Kahler, E.M. Simpanen, D. Operario, User-centered, interaction design research approaches to inform the development of health risk behavior intervention technologies, Internet Interventions. 15 (2018) 1–9. https://doi.org/10.1016/j.invent.2018.10.002.

[20] U.T. Jefferson, I. Zachary, W. Majee, Employing a User-Centered Design to Engage Mothers in the Development of a mHealth Breastfeeding Application, CIN: Computers, Informatics, Nursing. 37 (2019). https://journals.lww.com/cinjournal/Fulltext/2019/10000/Employing_a_User_Centered_Design_to_Engage_Mothers.5.aspx.

[21] J. Brooke, SUS-A quick and dirty usability scale, CRC Press, 1996.

[22] L. Zhou, D. DeAlmeida, B. Parmanto, Applying a User-Centered Approach to Building a Mobile Personal Health Record App: Development and Usability Study, JMIR Mhealth Uhealth. 7 (2019) e13194. https://doi.org/10.2196/13194.

[23] J.R. Lewis, IBM computer usability satisfaction questionnaires: Psychometric evaluation and instructions for use, International Journal of Human–Computer Interaction. 7 (1995) 57–78. https://doi.org/10.1080/10447319509526110.

[24] C. Pricilla, D.P. Lestari, D. Dharma, Designing Interaction for Chatbot-Based Conversational Commerce with User-Centered Design, in: 2018 5th International Conference on Advanced Informatics: Concept Theory and Applications (ICAICTA), IEEE, 2018: pp. 244–249. https://doi.org/10.1109/ICAICTA.2018.8541320.

[25] R. Vilardaga, J. Rizo, E. Zeng, J.A. Kientz, R. Ries, C. Otis, K. Hernandez, User-Centered Design of Learn to Quit, a Smoking Cessation Smartphone App for People With Serious Mental Illness, JMIR Serious Games. 6 (2018) e2. https://doi.org/10.2196/games.8881.

[26] C. LeRouge, J. Ma, S. Sneha, K. Tolle, User profiles and personas in the design and development of consumer health technologies, International Journal of Medical Informatics. 82 (2013) e251–e268. https://doi.org/https://doi.org/10.1016/j.ijmedinf.2011.03.006.

[27] W.-T. Hsieh, Y.-C. Su, H.-L. Han, M.-Y. Huang, A Novel mHealth Approach for a Patient-Centered Medication and Health Management System in Taiwan: Pilot Study, JMIR MHealth and UHealth. 6 (2018) e154–e154. https://doi.org/10.2196/mhealth.9987.

[28] P.P. Morita, M.S. Yeung, M. Ferrone, A.K. Taite, C. Madeley, A. Stevens Lavigne, T. To, M.D. Lougheed, S. Gupta, A.G. Day, J.A. Cafazzo, C. Licskai, A Patient-Centered Mobile Health System That Supports Asthma Self-Management (breathe): Design, Development, and Utilization, JMIR Mhealth Uhealth. 7 (2019) e10956. https://doi.org/10.2196/10956.

[29] S.Y. Crawford, A.D. Boyd, A.K. Nayak, N.K. Venepalli, S. Cuellar, S.M. Wirth, G.I.-H. Hsu, Patient-centered design in developing a mobile application for oral anticancer medications, Journal of the American Pharmacists Association. 59 (2019) S86-S95.e1. https://doi.org/10.1016/j.japh.2018.12.014.

[30] C.I. Reis, C.S. Freire, J. Fernández, J.M. Monguet, Patient Centered Design: Challenges and Lessons Learned from Working with Health Professionals and Schizophrenic Patients in e-Therapy Contexts, in: M.M. Cruz-Cunha, J. Varajão, P. Powell, R. Martinho (Eds.), ENTERprise Information Systems, Springer Berlin Heidelberg, Berlin, Heidelberg, 2011: pp. 1–10.

[31] D. Johnson, S. Deterding, K.-A. Kuhn, A. Staneva, S. Stoyanov, L. Hides, Gamification for health and wellbeing: A systematic review of the literature, Internet Interventions. 6 (2016) 89–106. https://doi.org/https://doi.org/10.1016/j.invent.2016.10.002.

[32] N.P. Cechetti, E.A. Bellei, D. Biduski, J.P.M. Rodriguez, M.K. Roman, A.C.B. De Marchi, Developing and implementing a gamification method to improve user engagement: A case study with an m-Health application for hypertension monitoring, Telematics and Informatics. 41 (2019) 126–138. https://doi.org/https://doi.org/10.1016/j.tele.2019.04.007.

[33] J. Hamari, Transforming homo economicus into homo ludens: A field experiment on gamification in a utilitarian peer-to-peer trading service, Electronic Commerce Research and Applications. 12 (2013) 236–245. https://doi.org/https://doi.org/10.1016/j.elerap.2013.01.004.

[34] L. Sardi, A. Idri, J.L. Fernández-Alemán, A systematic review of gamification in e-Health, Journal of Biomedical Informatics. 71 (2017) 31–48. https://doi.org/https://doi.org/10.1016/j.jbi.2017.05.011.

[35] M. Portela, C. Granell-Canut, A New Friend in Our Smartphone?: Observing Interactions with Chatbots in the Search of Emotional Engagement, in: Proceedings of the XVIII International Conference on Human Computer Interaction, ACM, New York, NY, USA, 2017: pp. 48:1--48:7. https://doi.org/10.1145/3123818.3123826.

[36] M. Siutila, The gamification of gaming streams, in: GamiFIN, 2018.

[37] DeepBot, (n.d.).

[38] PhantomBot, (n.d.).

[39] R.M. Puhl, C.A. Heuer, Obesity stigma: important considerations for public health, American Journal of Public Health. 100 (2010) 1019–1028. https://doi.org/10.2105/AJPH.2009.159491.

[40] G. van Rossum, Python tutorial, technical report cs-r9526, Amsterdam, n.d.

[41] R. Rivest, The MD5 Message-Digest Algorithm, (1992).

[42] I. Trinidad Rodríguez, J. Fernández Ballart, G. Cucó Pastor, E. Biarnés Jordà, V. Arija Val, Validación de un cuestionario de frecuencia de consumo alimentario corto: Reproducibilidad y validez, Nutricion Hospitalaria. 23 (2008) 242–252. http://www.nutricionhospitalaria.com/mostrarfile.asp?ID=4035.

[43] A. Bach-Faig, E.M. Berry, D. Lairon, J. Reguant, A. Trichopoulou, S. Dernini, F.X. Medina, M. Battino, R. Belahsen, G. Miranda, L. Serra-Majem, Mediterranean diet pyramid today. Science and cultural updates., Public Health Nutrition. 14 (2011) 2274–2284. https://doi.org/10.1017/S1368980011002515.

[44] A. Fadhil, G. Schiavo, Y. Wang, B.A. Yilma, The Effect of Emojis when Interacting with Conversational Interface Assisted Health Coaching System, in: Proceedings of the 12th EAI International Conference on Pervasive Computing Technologies for Healthcare, ACM, New York, NY, USA, 2018: pp. 378–383. https://doi.org/10.1145/3240925.3240965.

[45] J. Koivisto, J. Hamari, The rise of motivational information systems: A review of gamification research, International Journal of Information Management. 45 (2019) 191–210. https://doi.org/https://doi.org/10.1016/j.ijinfomgt.2018.10.013.

[46] M. Schmidt-Kraepelin, S. Thiebes, S. Stepanovic, T. Mettler, A. Sunyaev, Gamification in Health Behavior Change Support Systems - A Synthesis of Unintended Side Effects, 2019.

[47] T.L. Smestad, Personality matters! Improving the user experience of chatbot interfaces-personality provides a stable pattern to guide the design and behaviour of conversational agents, NTNU. (2018).

[48] E. Go, S.S. Sundar, Humanizing chatbots: The effects of visual, identity and conversational cues on humanness perceptions, Computers in Human Behavior. 97 (2019) 304–316. https://doi.org/https://doi.org/10.1016/j.chb.2019.01.020.

[49] T. Rietz, I. Benke, A. Maedche, The Impact of Anthropomorphic and Functional Chatbot Design Features in Enterprise Collaboration Systems on User Acceptance, in: 2019.

[50] E. Heller, Psicología del color : cómo actúan los colores sobre los sentimientos y la razón, (2004).

[51] B. Bazán, La conexión emocional con el color. Los colores que más y menos gustan en España y sus significados, Revista Sonda. Investigación En Artes y Letras. 7 (2018) 275–290.

[52] A. Bangor, P. Kortum, J. Miller, Determining What Individual SUS Scores Mean: Adding an Adjective Rating Scale, J. Usability Stud. 4 (2009) 114–123.
