# Supplementary material for: A User-Centered Chatbot (Wakamola) to Collect Linked Data in Population Networks to Support Studies of Overweight and Obesity Causes: Design and Pilot Study
Source: JMIR Med Inform. 2021 Apr 14;9(4):e17503. doi: 10.2196/17503 (PMC8087340; doi:10.2196/17503)
Supplement: Multimedia Appendix 2 [file medinform_v9i4e17503_app2.docx]

**Multimedia Appendix 2.** Wakamola chatbot.


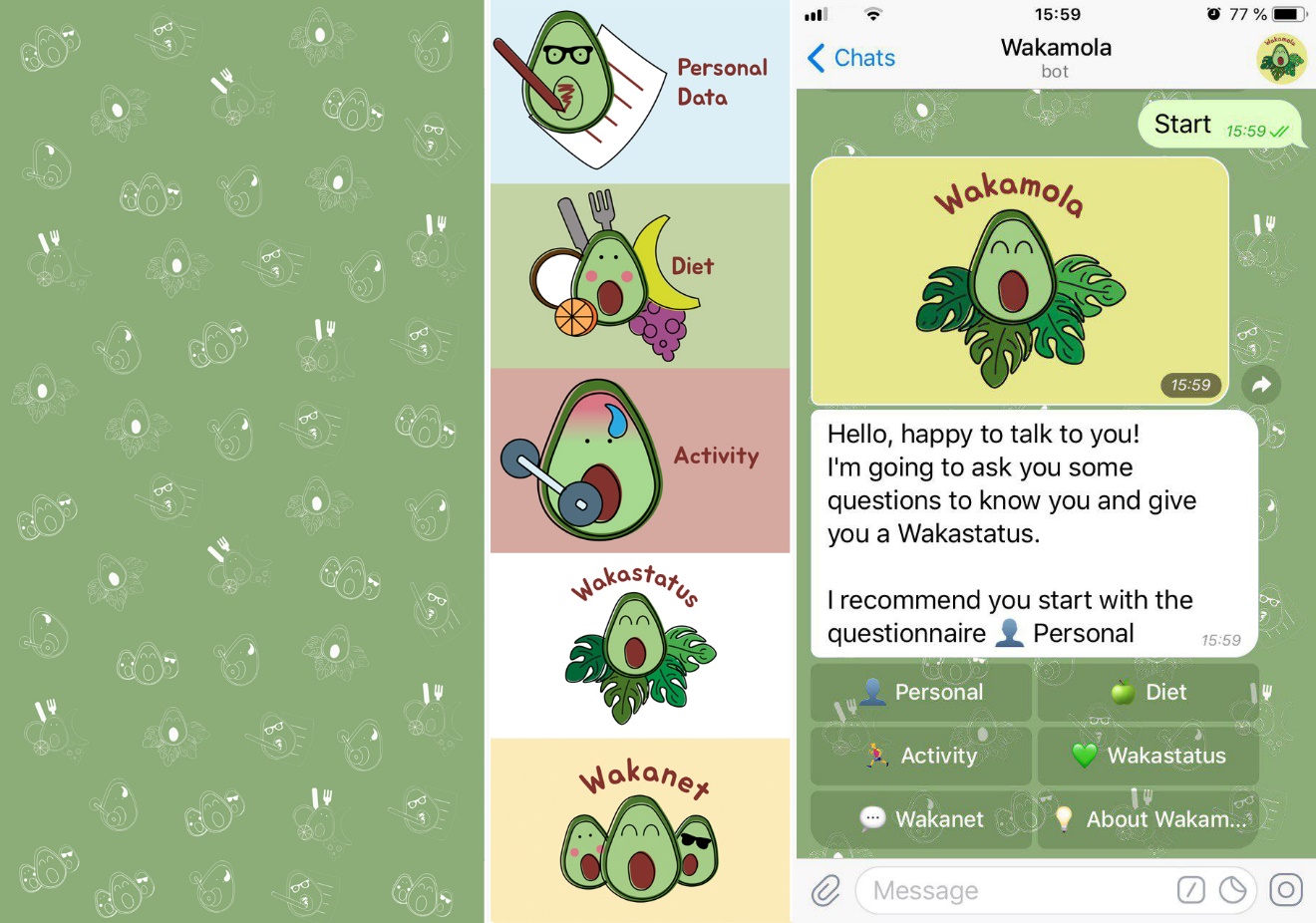


Figure S1. Wakamola´s background theme (left), stickers designed for each section (center) and a screenshot of Chatbot´s main menu (right).


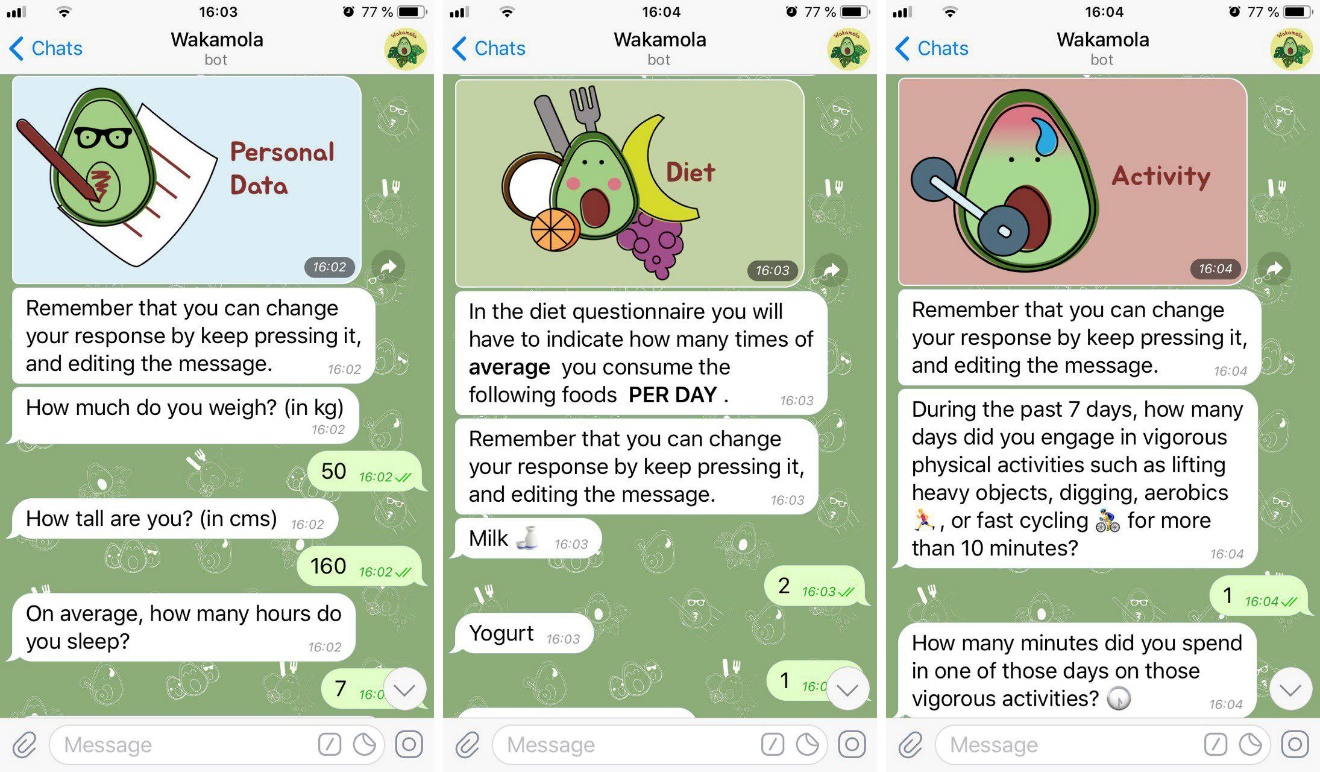


Figure S2. Wakamola screenshots of personal, diet, activity sections.


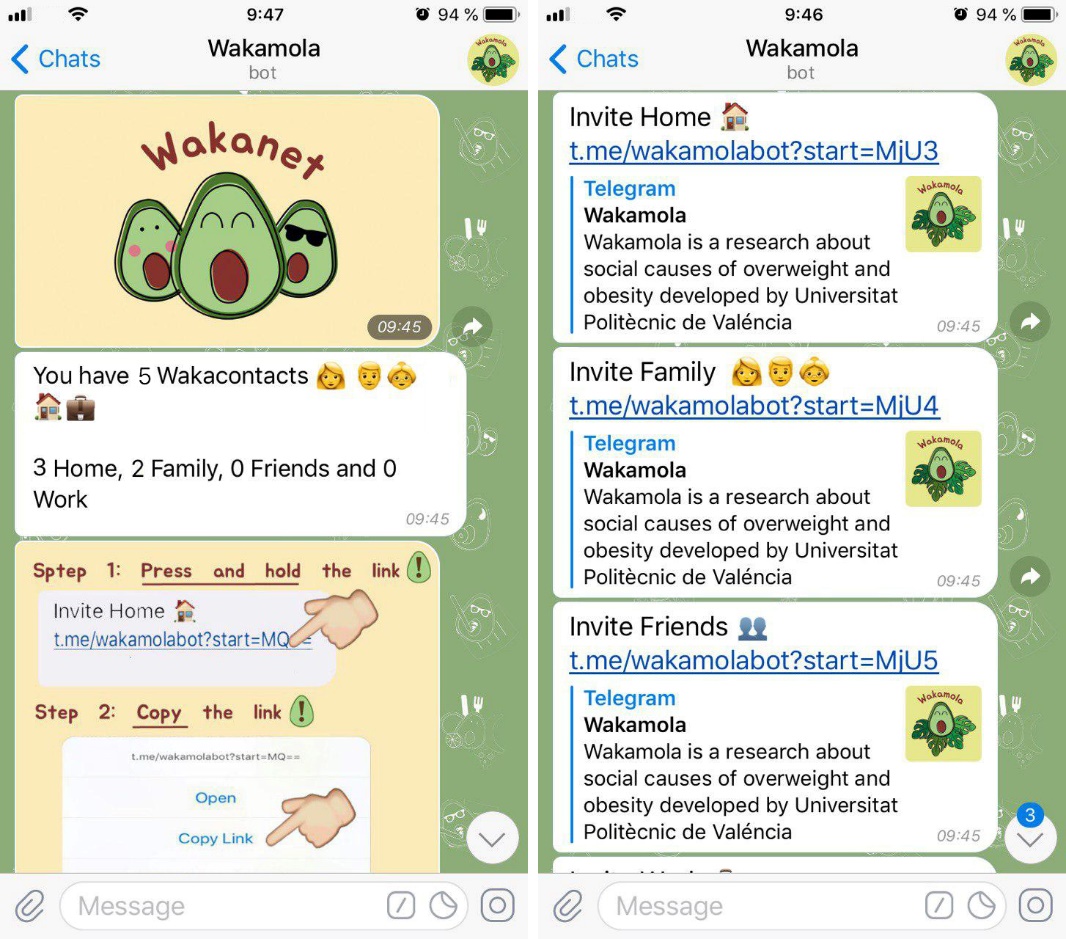


Figure S3. Wakanet section screenshots; (left) message about a number of contacts in total and by groups, and message to help sharing process; (right) messages to be shared to build the social network.


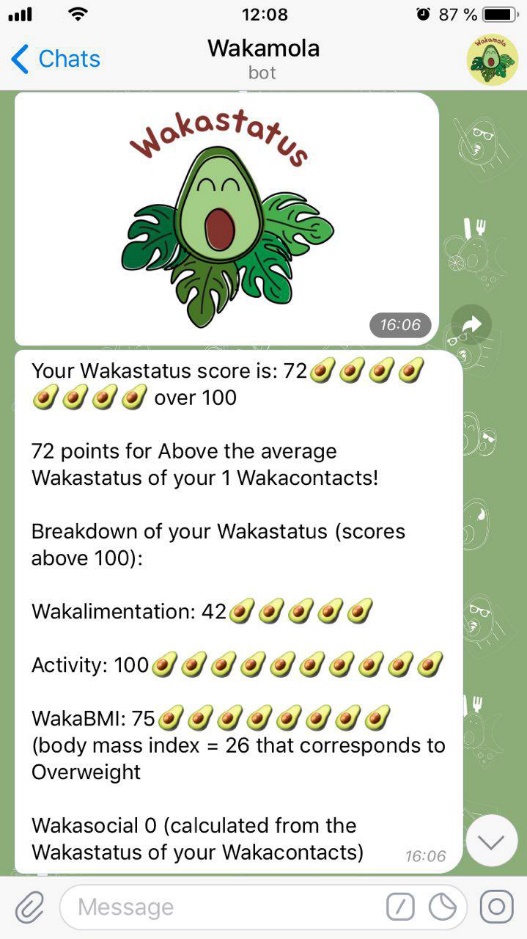


Figure S4. Wakastatus section screenshot.


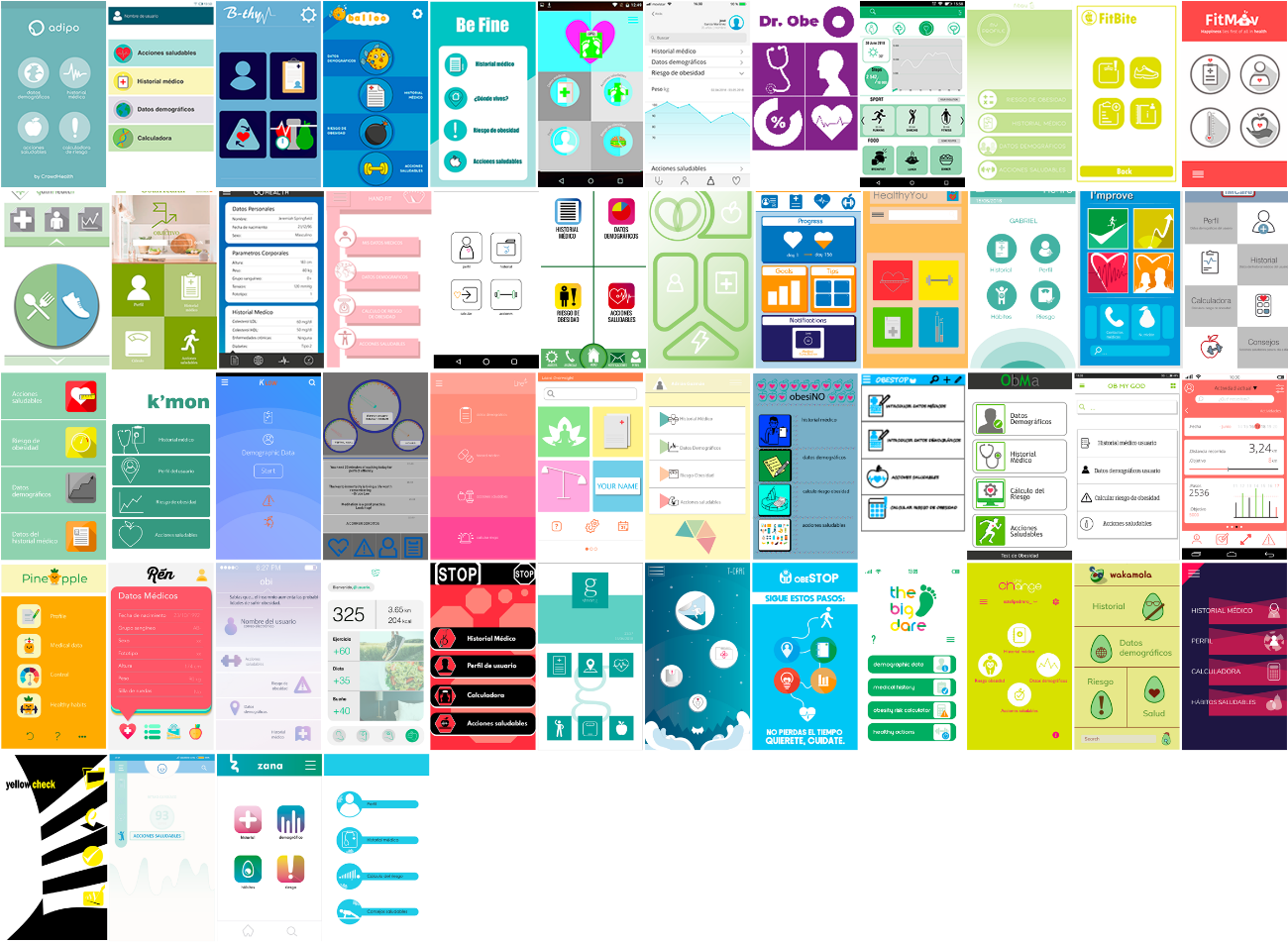


Figure S5. Collage of fifty-two wireframes proposed by one hundred and fifty design students from the Universitat Politècncia de València for an app in the field of obesity and overweight prevention.
